# Incorporating a gender lens into nutrition and health-related policies in Fiji: analysis of policies and stakeholder perspectives

**DOI:** 10.1186/s12939-022-01745-x

**Published:** 2022-10-15

**Authors:** Briar L. McKenzie, Gade Waqa, Sarah Mounsey, Claire Johnson, Mark Woodward, Kent Buse, Anne Marie Thow, Rachael McLean, Jacqui Webster

**Affiliations:** 1 The George Institute for Global Health, University of New South Wales, Level 5, 1 King St, Newtown, NSW 2042 Australia; 2 C-POND, Fiji National University, Suva, Fiji; 3 Menzies Centre for Health Policy and Economics, Charles Perkins Centre, The University of Sydney, Sydney, Australia; 4 The George Institute for Global Health, Imperial College London, London, UK; 5 Department of Preventive and Social Medicine, University of Otago, Dunedin, New Zealand

**Keywords:** Gender, Food policy, Non-communicable diseases, Nutrition interventions, Fiji

## Abstract

**Background:**

Gender equality, zero hunger and healthy lives and well-being for all, are three of the Sustainable Development Goals (SDGs) that underpin Fiji’s National Development Plan. Work towards each of these goals contributes to the reduction of non-communicable diseases (NCDs). There are gender differences in NCD burden in Fiji. It is, however, unclear whether a gender lens could be more effectively included in nutrition and health-related policies.

**Methods:**

This study consisted of three components: (i) a policy content analysis of gender inclusion in nutrition and health-related policies (*n* = 11); (ii) policy analysis using the WHO Gender Analysis tool to identify opportunities for strengthening future policy; and (iii) informant interviews (*n* = 18), to understand perceptions of the prospects for gender considerations in future policies.

**Results:**

Gender equality was a goal in seven policies (64%); however, most focused on women of reproductive age. One of the policies was ranked as gender responsive. Main themes from key informant interviews were: 1) a needs-based approach for the focus on specific population groups in policies; 2) gender-related roles and responsibilities around nutrition and health; 3) what is considered “equitable” when it comes to gender, nutrition, and health; 4) current considerations of gender in policies and ideas for further gender inclusion; and 5) barriers and enablers to the inclusion of gender considerations in policies. Informants acknowledged gender differences in the burden of nutrition-related NCDs, yet most did not identify a need for stronger inclusion of gender considerations within policies.

**Conclusions:**

There is considerable scope for greater inclusion of gender in nutrition and health-related policies in Fiji. This could be done by: 1) framing gender considerations in ways that are actionable and inclusive of a range of gender identities; 2) undertaking advocacy through actor networks to highlight the need for gender-responsive nutrition and health-related policies for key stakeholder groups; 3) ensuring that data collected to monitor policy implementation is disaggregated by sex and genders; and 4) promoting equitable participation in nutrition related issues in communities and governance processes. Action on these four areas are likely critical enablers to more gender equitable NCD reduction in Fiji.

**Supplementary Information:**

The online version contains supplementary material available at 10.1186/s12939-022-01745-x.

## Background

Diet-related non-communicable diseases (NCDs) are the leading causes of death globally [1]. The burden of NCDs is increasing among women and increasing disproportionately among women in low- and middle-income countries (LMICs) compared to women in high-income countries [2]. The Pacific Island nations experience some of the highest risks and burdens of NCDs, including a high prevalence of malnutrition [3].Therefore, the implementation of effective policies to address the burden of diet related disease is a priority for governments in the region [4].

Fiji is one of the larger Pacific Island Nations, with a population of approximately 900,000 people, and is classified as a middle-income country [5]. Within this population, 42% of women and 22% of men live with obesity [6]. In 2011, Fijian women were found to be more likely than men to have three or more of five key NCD risk factors: current smoking, consuming less than five servings of fruit and vegetables per day; low level of physical activity; overweight; and/or raised blood pressure [6]. Further, a high prevalence of iron deficiency anaemia, particularly in women, was identified in the 2014 National Nutrition Survey [7].

There are both biological (sex) and social (gender) reasons for differences in dietary intake, and diet-related disease risk between women and men. At a biological level there are some sex differences in nutrient requirements. Women of reproductive age, or who are pregnant, have different requirements for some micronutrients to men. Men generally require a higher intake of energy (and corresponding macronutrients) due to their higher lean body mass than women [8]. Previous research has illustrated how gender roles and responsibilities can influence food provision and the health of families [9–11]. In many countries, including Fiji, women tend to be responsible for the bulk of the reproductive labour and care defined as childrearing, cooking, cleaning, and community work [12]. This means that women can act as the “gate keepers” for food provision, and that gender identity may impact on diet related behaviours which may influence health outcomes. Therefore, gendered norms and practices (including marketing and promotion) concerning nutrition ought to be considered in food policy formulation and implementation [13].

Despite evidence of gender differences in dietary intake and diet-related disease risk, it is unclear how comprehensively gender has been included in nutrition and health related policies in Fiji. Gender- based analyses acknowledge gender-based inequalities and focus on assessing policies or programs so that they can be designed to address these inequalities [14].Therefore, responding to gender issues in nutrition and health-related policies could also contribute towards more effective implementation of the Sustainable Development Goals 2, “Zero hunger”, 3, “Good Health and Well-being” and 5, “Gender Equality” [15]. The aims of this study were to explore: to what extent nutrition and health- related policies in Fiji incorporate gender considerations and; key stakeholders’ perceptions on the importance and feasibility of, and opportunities for, incorporating gender-responsive measures into future polices.

## Methods

This study is a component of a broader program of work, that aims to support the scale-up of food policy interventions in Pacific Island Countries. The broader work was co-designed by researchers at Fiji National University, The George Institute for Global Health, University of Sydney, and Deakin University [16].

The approach to the present study is based broadly on the ideas of feminist theory, as we aimed to assess the inclusion of gender as a construct in nutrition related policy, and ultimately, the ability of these policies to contribute towards gender equity in health outcomes (by reducing diet-related disease risk in a gender equitable manner) [17].

### Terminology

Given the aims of our paper, we use the term “gender” throughout [18]. However, we often discuss gender in a binary way (women and men). This is because this is how gender is referred to in the policies reviewed, and how gender was identified and discussed by informants. We do, however, acknowledge that gender is non-binary. Where policies referred to sex instead of gender, we have used the term “sex”, as defined in the included policies. The term gender identity is used in this paper to refer to a “person’s deeply felt, internal and individual experience of gender” [18]. The terms gender “equality” and “equity” are also used in this paper. We have interpreted these terms based on definitions used by the World Health Organization [18] and by Global Health 50/50 [19]. Gender equality refers to equal conditions and opportunities to be healthy regardless of gender [18, 19]. Gender equity in health then refers to addressing the different health needs of people according to their gender [19].

We conducted three analyses: (1) an analysis of existing policy content; (2) an analysis of current policy strengths and opportunities; and (3) an analysis of stakeholder perceptions around gender inclusion in policies as follows:

### The inclusion of gender in existing policy content

A desk-based review of policy content was conducted between March and October 2020. We identified nutrition and health-related policies through: (1) online searches of Government and relevant within-country organization websites; (2) snowballing of relevant information from the initial search (for example, referenced guidelines, strategies, policies and action plans); and (3) direct requests to government ministries (ministries of health, industry and trade, agriculture, women, children and poverty alleviation, education and economy). Policies were included if they had relevance to nutrition and health-related issues in Fiji and/or if the policies would impact the formation of nutrition and health-related policies (for example, fiscal policies were considered as they provide important information on where and how funds are spent in Fiji).

Whether, and how, gender or sex was included in the policies, in terms of policy goals and activities, representation of women and men (that is in terms of population beneficiary group), and consideration of evidence that includes gender or sex-disaggregated data, was extracted into a matrix (supplementary table 1). Information on each policy’s objectives and overarching activities was also included.

### Analysis of current policy content—opportunities for strengthening

We analysed the gender inclusions with reference to global “best practice”. To do this, we utilised a gender matrix, building on ideas from the World Health Organization’s Gender Assessment Tool [20, 21] (supplementary table 1). This tool is based on an assessment of policy content, including how terminology is used, along with the extent to which the content is gender sensitive, specific or transformative, or conversely the extent to which the content is gender-blind or gender-unequal [20]. We extracted relevant data related to each criterion, in order to identify where opportunities and strengths are within the policy. We also identified common opportunities for strengthening gender-responsiveness across the assessed policies, to inform future efforts at enabling more gender-responsive nutrition and health-related policy.

### Stakeholder analysis

Semi-structured interviews were conducted with 18 informants in Fiji, between May and August 2020, via Zoom. The informants consisted of six government, five development partners, four private sector and three civil society representatives. Eight men and 10 women were interviewed. Interviews were led by BM or SM, with support from GW, and all interviews were conducted in English. Questions relating to gender, and the incorporation of gender into nutrition and health-related policy formed one section of the interview tool for a related study on strengthening the implementation of food policies in Fiji [16, 22].

The key informants approached to take part in this study were identified via the policy documents, defined as ‘*actors who have an interest in the issue under consideration, who are affected by the issue or – because of their position – have or could have an influence on the decision-making and implementation processes’* [23]. Given our aims, the focus was specifically on nutrition and NCD- related informants who have, or could have, an influence on nutrition and NCD-related decision making, as advised by local collaborators. The initial sample commenced with: 1) government agencies with responsibilities related to fiscal policy and/or nutrition (e.g. Ministries of Women and Poverty Alleviation, Finance, Industry/Commerce, Trade, Health, Agriculture); 2) food industry actors; and 3) civil society actors with an interest in health and/or food. Recruitment of interviewees was through formal (written) approaches to the heads of relevant agencies. Once approval was obtained, relevant departments were contacted to request interviews. At the end of each interview, we asked interviewees to identify further relevant interviewees (within and/or outside of their policy area).

A thematic analysis was conducted focusing on the perceived consideration/inclusion of gender in current nutrition and health-related polices, the perceived need to have a stronger and/or different focus on gender in policies, and the enablers and barriers to such inclusion. This analysis followed inductive coding (i.e. the coding of text that related to our research aims), along with deductive coding, based on the WHO gender assessment tool [20] and the FAO gender mainstreaming framework [24], Table 1. Both inductive and deductive coding was used to ensure that we captured themes that we had not pre-empted when we established this study, along with themes in line with the other aspects of our policy analysis (based on the WHO and FAO tools) [25]. Codes were then mapped to overarching themes (supplementary table 2). SM extracted high level gender-related codes (pulling gender-related information into broad gender codes), BM validated this coding and then conducted in-depth inductive and deductive analysis of the transcripts. The coding framework was discussed with GW, RM and JW with a particular focus on ensuring local cultural knowledge was prioritized. Inductive codes were discussed with GW, RM and JW as they were identified. NVivo software was used for transcribing the audio files (which were then validated). It was also used for analysis of the transcripts and data management.

**Table 1:** Coding framework

	**Codes**
**Deductive codes**, based on the World Health Organization gender assessment tool [20] and the Food and Agriculture Organization gender mainstreaming framework [21, 24]	Nutrition and the life cycle
	Obesity and nutrition
	Income generating activities and spending income on nutrition
	Local (food) culture and gender
	Rights-based perspective related to gender and nutrition
	Targeting in nutrition
**Inductive codes**, identified during thematic analysis of the transcripts	Gender specific needs and disease risk
	The need to focus on other 'vulnerable' groups
	Current considerations of gender in policy
	Barriers to the inclusion of gender
	Enablers to the inclusion of gender

Ethics approval was granted by the University of New South Wales (HC200055) and Fiji National University (CHREC ID 184.20).

## Results

### The inclusion of gender in existing policy content

Eleven policy documents were reviewed [26–37] (Table 2). Seven policy documents explicitly mentioned gender considerations within the policy goals or objectives. These included the National Development Plan [26], the Strategic Plan [28], the draft Food and Nutrition Security policy [29], the Wellness Policy [30], the Fijian Trade Policy Framework [31], the Supplement to the Budget [36, 37], and the National Gender Policy [33]. Of these seven policies, two had explicit gender considerations that were broader than a focus on women. Specifically, the Wellness Policy stated “The policy will ensure that men, women, boys and girls are considered equally in the planning and implementation processes of all Wellness initiatives and programs” [30]. Also, the National Gender Policy stated “The overall goal of this policy is to promote gender equity, equality, social justice, and sustainable development in the Republic of Fiji. The Government of Fiji is committed to removing gender inequality in Fiji” [33]. In general, across policies, men and women were represented collectively. One exception was the classification of pregnant women as a vulnerable group in the Strategic Plan [28]. Five of the policies that had explicit gender considerations also referenced gender-related and/or specific evidence (generally as background to the formation of explicit gender goals or activities) [26, 28, 29, 31, 33]. In terms of policy development, four of these documents provided information on the consultation process [27, 28, 30, 33]. However, gender of participants in the consultation process was not defined.

**Table 2:** Description of gender inclusion in policies

	Whole of Government Plan	Health				Trade	Agriculture	Gender	Education		Economy
Included policies	5-Year & 20-Year National Development Plan Main author(s): Ministry of Economy Dates: 2017—2036 [26]	Non-Communicable Diseases Strategic Plan Main author(s): Ministry of Health, Australian Aid, C-POND Dates: 2015–2019 [27]	Strategic Plan Main author(s): Ministry of Health Dates: 2020 – 2025 [28]	Draft Fiji Policy on Food and Nutrition Security (FPFNS) Main author(s): Ministry of Health, Ministry of Agriculture Dates: 2018–2022 (Note, not endorsed) [29]	Wellness Policy Main author(s): Ministry of Health Dates: 2015 [30]	Fijian Trade Policy Framework Main author(s): Ministry of Industry, Trade and Tourism Dates: 2015–2025 [31]	Fiji 2020 Agriculture Sector Policy Agenda Main author(s): Ministry of Agriculture, and FAO Dates: 2014–2020 [32]	Fiji National Gender Policy Main author(s): Ministry for Social Welfare, Women & Poverty Alleviation Dates: 2014 (to be reviewed every 4 years) [33]	Fiji School Health Policy Main author(s): Ministry of Health and Ministry of Education, Heritage & Arts Dates: 2016 (reviewed every 2 years) [34]	Policy on Food and School Canteens Main author(s): Ministry of Education, Heritage & Arts Dates: 2017 [35]	and Fiscal Update—Supplement to the 2019–2020 budget address Main author(s): Ministry of Economy Dates: 2019–2020 [36, 37]
Explicit consideration of gender in policy goals and activities?	Yes	No	Yes	Yes	Yes	Yes	No	Yes	No	No	Yes
How are men, women and other groups represented?	Collectively	Collectively	Pregnant women are mentioned as a "vulnerable" group	Collectively	Collectively	Not differentiated	Not differentiated	Collectively, although specific aims focus on different groups of women and men, girls and boys	Not differentiated	Not differentiated	Not differentiated
Is there consideration of evidence that includes gender?	Yes	No	Yes	Yes	No	Yes	No	Yes	No	No	No
Information on consultation process and gender of participants?	Not covered	Outline of the consultation process; the workshop summary gives a list of names with females represent approximately 50% of the participants	Consultation process described, but details based on gender not provided	Not covered	The consultation process is described in the Annex, and the contributing senior officials were named. (but gender not defined)	Not covered	Not covered	The consultation process is described. 37 out of the 55 stakeholders consulted on the policy were female (71%)	Not covered	Not covered	Not covered

### Analysis of current policy content

**Fig. 1:**
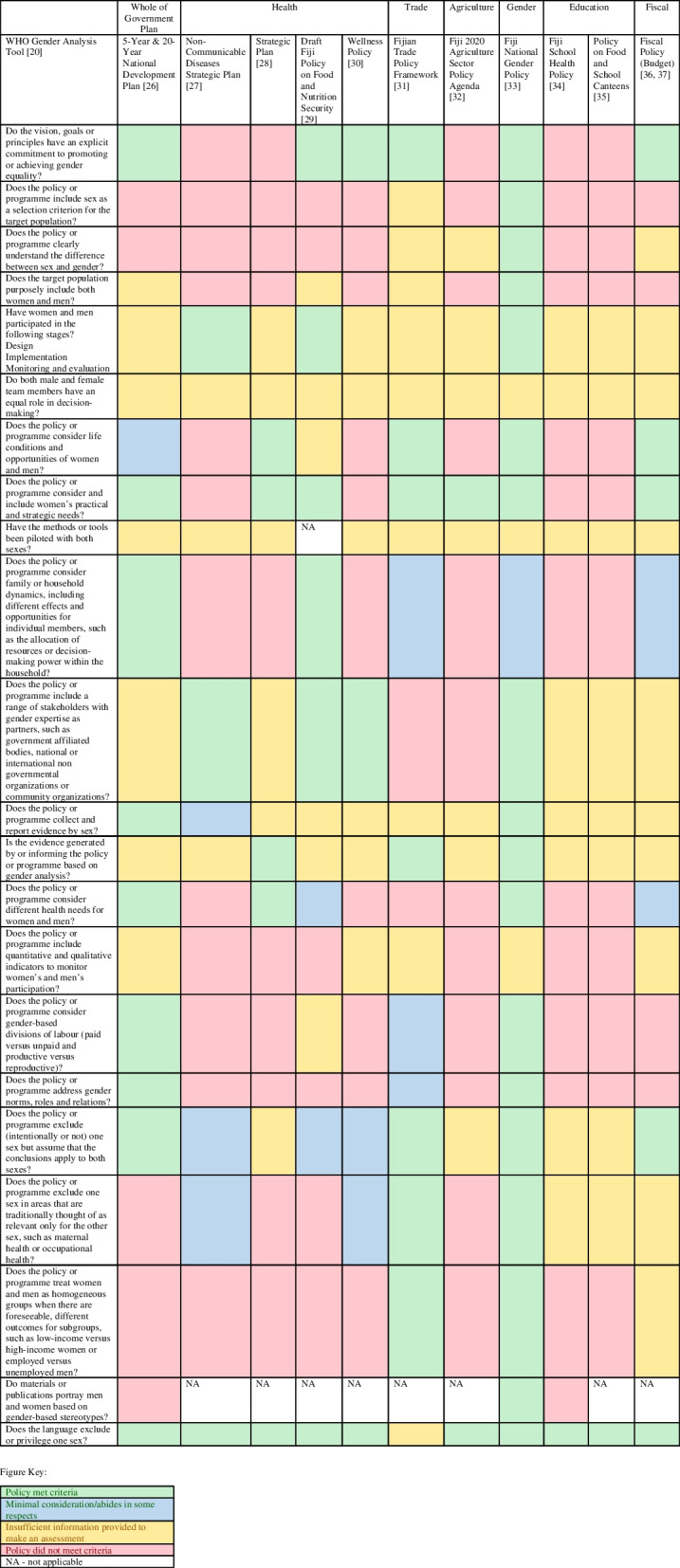
Gender analysis of diet related policies in Fiji, using the World Health Organization Gender Analysis Tool

Figure 1 shows the assessment of the 11 nutrition and health-related policies against the WHO Gender Assessment Tool, with rationale for categorisation provided in Supplementary Table 1. Of the six policies that had explicit consideration and commitment to promoting or achieving gender equality, the Fiji National Gender Policy stated that programs or activities should include sex as a selection criterion for target populations and purposely include both women and men [33]. Only the Gender Policy clearly defined what was meant by the terms “sex” and “gender”.

Most policies did not include considerations of life conditions and opportunities for women and men, nor did they reflect on family and household dynamics, opportunities, resource allocation or decision-making power within households, that might impact on attainment of policy goals across population groups. Exceptions to this included the National Development Plan, which stated that *"These reforms provide a platform for equality where both men and women can enjoy the benefits of employment and conditions conducive to productivity and prosperity for all*" and “*It is expected that home duties in caring for children and household work will be shared by the spouse or partner”* [26]. Conversely, six policies did have considerations related to women’s practical and strategic needs. For example, the Agriculture Sector Policy included an objective to create an investment fund for "retirees, women, and youth", to help attract these groups to the farming industry [32]. The NCD Strategic Plan [27], the draft Food and Nutrition Security Policy [29] and the Gender Policy [33], clearly stated that both women and men had been involved in policy development, and that both men and women would be involved in policy implementation, monitoring and evaluation.

According to this review, the most gender responsive policy was the Fiji National Gender Policy [33]. The Strategic Plan [28] and the Fijian Trade Policy Framework [31] both referred to the National Gender Policy. However, how various elements of the gender policy would be incorporated into the subsequent action plans was not specified. There are identified opportunities for policy strengthening across government ministries (Fig. 1).

### Stakeholder perceptions

Key themes and illustrative quotes are presented in Supplementary Table 2. The main themes identified from the WHO and FAO frameworks included: income-generating activities and spending income on nutrition; local (food) culture and gender; nutrition and the lifecycle; obesity and nutrition; rights-based perspective related to gender and nutrition; and targeting in nutrition. Themes identified inductively, during the coding process included: barriers and enablers to having gender considerations in nutrition and health-related policies; gender-specific needs and disease risk; current considerations of gender in policy; and the need to focus on other ‘vulnerable’ groups (rather than women, or gender considerations more broadly). These themes were grouped to aid interpretation (supplementary table 2):

#### Perceptions on gender, health, and nutrition – a needs-based approach for the focus on specific groups in nutrition and health related policy

##### Gender-specific needs and disease risk

Most of the discussion on gender-specific needs and disease risk focused on women of reproductive age and iron deficiency anaemia. However, there was acknowledgement of a higher incidence of premature death due to NCDs in men in Fiji.

*“Yeah, I think anaemia is towards women more than men. Women outlive men in Fiji. Men die more earlier to NCDs—high blood pressure is also on women. Also obesity in children” – Government, W.*

##### Nutrition and the lifecycle

Any discussion around nutritional needs and the lifecycle was exclusively focused on women of reproductive age, including women who were pregnant or breastfeeding. In general, this discussion also focused on iron-deficiency anaemia, and the higher risk that women of reproductive age have for this condition.

##### Obesity and nutrition

There was a consensus that, while obesity was prevalent across genders in Fiji, there was a higher prevalence of women living with overweight and obesity (supported by the most recent WHO STEPs survey [6]). How to address this gender difference was not discussed in depth. Interviewees suggested that the burden of obesity in the region needed to be addressed more generally, not by a gender- or sex-specific response.

*“There is clear evidence that, one, in terms of overweight and obesity, then one is bigger than the other. But in terms of policy, we need to talk more about that instead of if we need to be more gender specific…” – Government, M.*

#### Perceptions on gender-related roles and responsibilities around nutrition and health

##### Income-generating activities and spending income on nutrition

Interviewees reflected that there are increasing numbers of women in the formal paid workforce, particularly in the urban region of Suva. They reflected that this shift has corresponded with increased consumption of convenience foods, highlighting that women maintain responsibility for their family’s nutritional needs.

*“And so people are working longer hours than women who, like my mom, was a housewife. And so most of the wives now are no longer the housewives. They are all part of the mad rat race and so getting home to cook the food, and when I say cook the food, it's not only woman, it's also men who ought to cook the food” – Private sector, M.*

##### Food culture and gender

There was a lot of discussion around culture, gender, and food in Fiji. Interviewees reflected on a strong sense of culture in Fiji, and how there are traditional gender roles around food, although it was highlighted that the gender roles and expectations around food were changing.

*“We engineer the thought process [where] women are the nurturers, the feeders, and sometimes they give everything and there's nothing left for them.” – Civil society, W.*

Interviewees did not, however, uniformly think that the relationship between culture and food would necessarily have an impact on diet quality. Additionally, most did not see the social and cultural norms around food being related to gender inequality.

*“I think that the diets, whether it's Indo-Fijian [or Fijian], they're similar, the families eat together. It's a very important ritual, the family meal … there are similar challenges across the board for both men and women and children as such, because … everyone does eat.”– Private sector, M.*

#### Perceptions on what is considered “equitable” when it comes to gender, nutrition, and health

##### Rights-based perspectives related to gender and nutrition

A range of rights-based perspectives on gender and nutrition were identified. Some interviewees suggested that, if they were to focus on gender, it could risk being at the expense of other “vulnerable” groups, such as children, or may lead to identification of relatively unimportant differences.

*“Because if we were to demarcate to between men and women, I mean, already we are pre-empting and we are differentiating… ‘This is the emphasis that we place on men and this is the emphasis that we place on women’” – Government, W.*

It was acknowledged that, traditionally, women prepare the food for the household, yet are often the last to eat, highlighting a cultural aspect that translates into gender inequality. Interviewees also highlighted a need to move on from putting all the responsibility of nutrition, health, and wellbeing of the family on women.

*“… No, I am trying also to get away from the idea that women should be responsible for their own health issues, but also for their kids health. Because then we place the whole responsibility of nutrition on women, which I feel that is very unfair.” – Development partner, W.*

#### Perceptions of current considerations of gender in nutrition and health-related policies and ideas for further gender inclusion

##### Current considerations of gender in policy

There were a range of views on the current considerations of gender in policies. Some interviewees stated that policies had more general aims, but that they did include programs focused specifically on women. Others suggested that gender was considered within the “vulnerable” groups’ category. There was also a perception that “vulnerability” should be a focus in policies, and that this may not always mean a focus on gender (women), but rather a focus on those with highest need, dependent on the policy focus.

*“I think it's addressed in the policy. There's this area around the, you know, the needs of the vulnerable groups in the population, so it's addressed in the nutrition policy.” – Development partner, W.*

##### Targeting nutrition

Most interviewees acknowledged that there were gender differences in diet- related disease risk, and or needs. However, views on the need to target nutrition and health-related policies or interventions were mixed, and there was a general lack of acknowledgement that people of a certain gender(s) may be overlooked when developing nutrition and health-related policy.

*“I think no. I mean… the priority should be both genders. Why only one?” – Civil society, W.*

*“Yes, because the requirement for a woman is different than compared to men. So, I believe that when making these policies, both genders could be considered.” – Private sector, W.*

#### Enablers and barriers to the inclusion of gender in nutrition and health-related policy

For interviewees who agreed that gender considerations should be evident in nutrition and health- related policies, a range of barriers and potential enablers to their inclusion were identified. Key barriers included a lack of: (i) awareness around the need for gender considerations (broader than women’s reproductive health); (ii) collaborative and multisectoral platforms; and (iii) disaggregated data for the identification and monitoring of gender-related needs. Further inclusion of the National Gender Policy in nutrition and health-related policies, multisectoral engagement (for example, building on expertise from the Ministry of Women), and making it standard practice to collect and make available gender-disaggregated data, were identified as key enablers.

*“… if you want to include gender into their [policies] we need to create that environment first… So that they'll be able to accept it and be able to go out and work on gender.” – Development partner, W.*

## Discussion

Progress towards, and achievement of, the SDGs are central to Fiji’s whole-of-government National Development Plan [26]. Given marked differences by sex and gender in diet-related disease risk and burden, there is a need for sex and gender considerations to be included across policies and sectors that deal with health-related issues. From our analysis, we found that, while gender was considered in a number of the policies, only one policy was assessed as gender responsive, and this was the National Gender Policy [33]. Most informants were ambivalent around the need for stronger inclusion of gender considerations, although there was an acknowledgement that there are sex and gender differences in diet-related disease burden in Fiji.

The Government of Fiji has demonstrated a commitment to gender equality, through its National Gender Policy. This policy is led by the Ministry of Women, Children and Poverty Alleviation, but with the aim to be cross cutting, applying to all government ministries, and with the overall goal to “promote gender equity, equality, social justice and sustainable development in the Republic of Fiji” [33]. The Policy, and achievements towards the policy goal, are reviewed every four years in line with the review process for the Convention on the Elimination of All Forms of Discrimination Against Women (CEDAW) [38]. Whilst the National Gender Policy was the only policy found to be gender responsive, it is encouraging that a number of the other policies refer to it, and key informants were aware of the policy. However, our analysis showed that there is a lack of detail in most policies concerning how the goal of gender equity could be achieved in terms of diet and related NCD burden. Training within government ministries could aid the tailoring of programs to ensure they are gender responsive, and to ensure that they include actionable steps towards the broader gender-related goals. Whilst there are related costs, research shows that the degree of budgetary commitment drives successful policy implementation, highlighting the need for advocacy targeted towards the Treasury and the Ministry of Economy [39].

Some informants identified differences by gender in the prevalence of obesity and the burden of premature NCD death in Fiji, yet they did not think that these differences would require more targeted programs. Most also did not link these differences with gender-related roles and/or responsibilities around nutrition, a consideration also lacking in the majority of the reviewed policies. There was an impression, from both women and men, that including gender considerations in policies could distract from other population groups with “higher needs”. Informants also identified actor-related barriers to the inclusion of gender considerations, including a lack of awareness around the need for gender considerations (broader than women’s reproductive health) and collaborative and multisectoral platforms. Globally, women are more likely to be affected by malnutrition (both under and over nutrition), and to be food-insecure [40]. Given the importance of gender in relation to the health of all people in Fiji, it is important for policy implementors to recognize that gender is a cross-cutting determinant of health [41]. There are key actors in this space in Fiji, including the Ministries of Women and Children and Poverty Alleviation, who lead the National Gender Policy, International Organizations including FAO and the Pacific Community, and NGOs including Diverse Voices, Action for Equality, FemLINKpacific and the Fiji Women’s Rights Movement. Evidence from both gender and nutrition-policy research shows that effective nutrition actor networks, spanning different sectors, can generate government commitment to issues [39, 42]. In the Pacific, evidence from regional policy forums on nutrition issues reflect the need for multi-sectoral response [43]. There are multi-sectoral working groups in Fiji that focus on specific nutrition issues (for example food labelling) or more broadly on NCD risk reduction. Therefore, there are opportunities to further strengthen the commitment to the inclusion of gender-related considerations in nutrition and health- related policies through pre-existing nutrition focused multi-sectoral platforms, with advocacy and awareness raising of nutrition-related stakeholders from key gender actors.

Several informants reflected on the changing work culture in Fiji, and its impacts on the roles and responsibilities of women. They reported a shift to more women working within the formal (paid) workforce, particularly in urban areas of Fiji. They suggested that these shifts have likely played a role in the changing burden of diet related disease, with a reliance on convenience foods which are generally highly processed, of low nutritional value, yet high in fat, salt and sugar [44]. This observation highlights that, even with more women in the workforce, women retain the responsibility for preparing food for their families. While the importance of addressing gender inequities in roles and responsibilities (including those relevant to nutrition) are highlighted in the National Development Plan, there are no identified mechanisms for addressing the negative implications [26]. An increasing proportion of women in the formal work force and changes in nutritional status of populations is not a new concept, nor is it unique to Fiji [45, 46]. Mkandawire et al. [21] conducted a gender assessment of Malawi’s National Nutrition Policy and Strategic Plan in 2016. While they identified that the policy was gender responsive, based on the WHO gender assessment and FAO tools, they proposed that there was a need to develop an environment that promoted boy’s and men’s participation in nutrition, including shopping, preparing and cooking food [21]. Informants in the present study similarly argued that nutrition should be viewed as a responsibility of men as well as women.

Across the policies reviewed (including the National Gender Policy), gender is referred to in binary terms, and there is no acknowledgement that gender is non-binary. Further, only a limited number of policies defined what they meant by “sex” or “gender”. There is a danger in referring to gender solely in binary terms, as this groups by femineity and masculinity, which can deepen existing stereotypes and corresponding roles and responsibilities [41]. Gender is about everyone; gender equality is everyone’s responsibility, and everyone benefits from gender equality. Yet, informants often responded to questions about gender and health only in terms of the implications for women of reproductive age, despite some acknowledgement that men should have a role in nutrition. Further, globally there have been calls to ensure that data is collected in a gender sensitive manner, and with the ability to disaggregate data by sex [42]. The availability of sex and gender data following policy implementation in Fiji will be crucial to understanding the influence of gender on policy implementation going forward.

A finding that underlies most of the above discussion points is that many of the informants interviewed were satisfied with the level of gender inclusion in nutrition and health-related policies in Fiji. We have discussed some factors related to this. However, there is a need to better understand why this is the case. It is possible that better evidence on the difference that gender sensitive policies can have on health outcomes is needed. It is also possible that there is a broader resistance to changing gender roles. As in most cultures, there are cultural norms and practices that define the role of women and men regarding food and nutrition in Fiji. For example, in iTaukei (Indigenous Fijian) culture, ideas around femininity and masculinity are largely based on Christian ideals of women being caring and nurturing and men being strong and being the head of the family [47]. While culture needs to be respected, it should not be at the expense of work towards gender equality [38]. It is possible that action in this area could stem from advocacy and mobilisation by feminist groups working towards gender equality more broadly, and that such action could trickle down to nutrition-related policy.

Considerable work is being done around women’s empowerment in Fiji. In 2019, the FAO in collaboration with the Pacific Community, conducted a country gender assessment of agriculture and the rural sector [48]. Key recommendations echo those of the present study, but focus specifically on women in rural settings. There is also extensive research around gender and fisheries in Fiji, with programs for women’s empowerment [49]. Gender-based work in agriculture and fisheries highlights the need for the representation of women in governance structures. This need was reflected by our policy review and interviews with informants. In 2020, representation of women in Parliament was 22% [50]. While this is positive, and is an increase on previous years, it shows that there is still scope for improvement. Globally, an initiative called “Global Food 50/50” has been introduced [51], which highlights how gender is reflected, or not, in the policies and practices of leading global food organisations. It aims to provide an accountability system for organizations to ensure gender-responsive programming, gender-equitable institutions and diversity of leadership within organizations [51]. Such tools could be used or adapted for the Fiji context.

### Strengths and limitations

To our knowledge, this is the first study to focus on gender considerations in nutrition and health-related policies in the Pacific Island region. We gained a range of insights and expertise from interviewees. However, some informants whose insights would likely have been beneficial, did not respond to requests for interviews. We were able to triangulate our findings from the different sections of our analysis and from different data sources which, in line with the literature discussed, informed our recommendations. The first author on this paper, who led the study and wrote the first draft of the article, is not from Fiji [52], they worked closely with the second listed author, who is a researcher from Fiji based at Fiji National University with expertise in qualitative research and policy analyses, who provided input and support on interpretation of findings. In terms of the stakeholder interviews, another limitation is that the questions on gender considerations made up one part of the overall interview guide for the larger study [22]. The interviews overall focused on nutrition and health-related policy and opportunities for scaling-up these policies. We propose that interviews focused specifically on gender considerations in policies could be conducted to gain more in-depth information particularly in relation to explanations for the lack of inclusion of gender considerations in policies. Further, other factors such as socioeconomic status, ethnicity, and region (urban compared to rural), as well as gender, could be explored in future analyses.

## Conclusion

Gender equality is a stated goal in several nutrition and health- related policies in Fiji, however, based on the WHO Gender analysis tool only one policy was ranked as gender responsive. The gender responsive policy, The National Gender Policy, aims to be cross-cutting across all government ministries. While this is a key strength in terms of accountability for monitoring progress towards gender equality in Fiji, we have identified opportunities to further strengthen gender considerations across nutrition and health- related policies. For the strengthening of gender considerations across policies, we suggest that gender equality advocates in civil society and nutrition focused organizations along with policy stakeholders in Fiji can focus on: 1) framing gender considerations in policies so that they are actionable and more inclusive of a range of gender identities; 2) undertaking advocacy through actor networks to highlight the need for gender-responsive nutrition and health- related policies across key stakeholder groups (including government, industry, civil society and development partners); 3) ensuring that data collected to monitor policy implementation is disaggregated by sex, and inclusive of gender identities; and 4) promoting equitable participation in nutrition-related issues at both a community and governance level. We propose that these steps will be crucial in the development of gender-responsive policies. However, future monitoring and evaluation of policy implementation will be needed to identify corresponding changes in practice.

## Supplementary Information

**Additional file 1: Supplementary table 1.** Policy content analysis and WHO gender analysis, data extraction.

**Additional file 2: Supplementary table 2. **Coding of stakeholder perspectives on gender considerations in nutrition and health related policy, and mapping to overarching themes.

## Data Availability

The datasets used and/or analysed during the current study are available from the corresponding author on reasonable request.
