# Supplementary material for: Incorporating a gender lens into nutrition and health-related policies in Fiji: analysis of policies and stakeholder perspectives
Source: Int J Equity Health. 2022 Oct 15;21:148. doi: 10.1186/s12939-022-01745-x (PMC9569111; doi:10.1186/s12939-022-01745-x)
Supplement: Supplementary file 2 — Additional file 2: Supplementary table 2. Coding of stakeholder perspectives on gender considerations in nutrition and health related policy, and mapping to overarching themes. [file 12939_2022_1745_MOESM2_ESM.docx]

**Supplementary table 2. Coding of stakeholder perspectives on gender considerations in nutrition and health related policy, and mapping to overarching themes.**

| Overarching themes | Codes | *Example 1* | *Example 2* |
| --- | --- | --- | --- |
| 1. Perceptions on gender, health and nutrition – a needs-based approach for the focus on specific groups in nutrition and health policy | Nutrition and the life cycle | Yeah, I think in terms of what we've said, we need to be what kind of food that we might need. Nutrition labelling on packaged food. I think to talk about women and their lifecycle, their needs to be more awareness on why women need more iron than men, and in the culture, where men eat fist then women. And how can we actually address, you know, the anaemia around child bearing age. Can we promote local, local food that can actually be used in like in hospital instead of tablets. It could be a substitute instead of taking iron tablets (Govt, W) | Well, I mean, with anaemia, you know, supplementation is generally only run for a certain population groups that have a high prevalence, which is set out in the Ministry of Health plan, but it's based on burden and of course, the burden tends to follow population subgroups but it's essentially about burden so you're and then for fortification, of course, when you're deciding fortification levels, then it's primarily around, you know, making sure that, you know, overdosing anybody and trying not to under dose anybody. But that's kind of part of the technical assessment rather than being stated directly in the policy. That's just how you assess fortification levels. So I guess it's the hidden subtext, but it's not an it's it's all related to burden and demanded intake rather than targeting gender specifically. (Development Partner, W) |
|  | Obesity and nutrition | There is clear evidence that, one, in terms of overweight and obesity, then one is bigger than the other. But in terms of policy, we need to talk more about that instead of if we need to be more gender specific because there's a clear difference in the gender. (Govt, M) | Obesity is also across genders, but it isn't just more prevalent in females. Definitely because of low physical activity and other factors and because obesity is more prevalent in females, they are pretty good chances that if we start screening and see through the population, that will be lowered by a steps survey because physical activity is low, consumption is high. Definitely. Prevalence would be different in both the sexes. (Govt, W) |
|  | Gender specific needs and disease risk | Yeah, I think anaemia is towards women more than men. Women outlive men in Fiji. Men die more earlier to NCDs - high blood pressure is also on women. Also obesity in children. (Govt, W) | For some of the NCDs it's across, and I think maybe where there is a difference between men and the ladies is cancer. Yeah. Ladies having cervical cancer, breast cancer. Well, it's men having prostate cancers is the only difference, but otherwise generally I feel it's the same across both. (Govt, M) |
| 2. Perceptions on gender-related roles and responsibilities around nutrition and health | Income generating activities and spending income on nutrition | It's the availability of processed foods. That's the problem now and so the Fijian is now becoming like the Australians of the past, the Americans of the past where everything is go, go, go, go. And so people are working longer hours than women who, like my mom, was a housewife. And so most of the wives now are no longer the housewives, they are all part of the mad rat race and so getting home to cook the food and when I say cook the food, it's not only woman it's also men who ought to cook the food. (Private sector, M) | Oh, yes. Well, yes. We are having an increase in number of women who are now working. For our food preparation culture is definitely changing. For example, for that time I'm really targeting workplace policies, because more of our women are working and we need to go into the workplace and talk to them about healthy workplace. That's a labor plan. Ministry of Education need to go in so that our children are informed. That's a sector. We need to bring it in. Then Ministry of Agriculture. And particularly Ministry of Trade, because I think that is the biggest....[?] it's the social mobilization. (Govt, M) |
|  | Local (food) culture and gender | Yeah. It'll be that, it also be on the misconceptions of culture, about women and also the access which is a big issues of women as well; so access to money to buy food, and I would say communication again. We engineer the thought process was it's like women are the nurturers, the feeders and sometimes they give everything and there's nothing left for them. (Civial Society, W) | Look, I don't think so. I think that the diets, whether it's Indo, Fijian. they're similar, the families eat together. It's a very important ritual, the family meal and that meal time. So there are similar challenges across the board for both men and women and children as such, because they everyone does eat. But the community and the community feel around meals is very important to Fijians. (Industry, M) |
| 3. Perceptions on what is considered “equitable” when it comes to gender, nutrition and health | Rights-based perspective related to gender and nutrition | Maybe yes. They may not, I'd say it ought to be whatever it is, it ought to be family centered or community centered so that everyone in the community or everyone in the household gets to benefit from. From this policies or this plan.. Because if we were to demarcate to between men and women, I mean, already we are pre-empting and we are differentiating that deal. This is the emphasis that we place on men and this is the emphasis that we place on women. I'd like to think that we need to -because we are exposed to the similar environment - I would think that we ought to be treated the same way as well. If there is a particular section of the society that we ought to place prominence on, I'd say you would have to be the children. Specifically the children under five. We need to work on that specific cohort. We need to work on them to allow that healthy transition throughout the life span. (Govt, W) | No, I am trying also to get away from the idea that women should be responsible for their own health issues, but also from their kids health. Because then we place the whole responsibility of nutrition on women, which I feel that is very unfair. (Development Partner, W) |
|  | The need to focus on other 'vulnerable' groups | Not particularly. I mean, I think the diets are broadly similar in both genders, in adults. I mean, I think what we're generally seeing is, you know, in young children, there's still quite significant issues of undernutrition and we're still seeing, you know, hospital admissions from malnutrition and that's never really gone away. We also see, you know, continuous problems of anaemia and things like that. But it's not particularly related to, you know, that small age group related and subgroup related like women during pregnancy, more might be anaemic, et cetera. But the dietary patterns, I think, you know, are slightly different between the two genders in adulthood. But it's not something that is huge in terms of impacting on nutritional status. (Development Partner, W) | Yes, this is target the sectors of the population that children under 5 and women of childbearing age. But then each stem of the policy talks to a different targeted sector of the population. So there is already that inclusion into the draft policy. But we'd like to, as I had mentioned, I'd like to see more of these interventions as family centered. Working on the entire family, that will rub off positively on the entire community. If we can work on the entire family into the interventions to benefit the entire family, that these families become success stories for the entire community. And the community is what that builds the entire nation. (Govt, W) |
| 4. Perceptions of current considerations of gender in nutrition and health related policies and ideas for further gender inclusion | Targeting in nutrition | Yes, because the requirement for a woman is different than compared to men. So I believe that when making these policies, both genders could be considered. (Private Sector, W) | I think it's important to have a gender aspect to it. Gender and also with children should be mentioned specifically because Fiji is very rich in its culture. So I think if you generalize, then it just falls through but there should be opportunities or women and opportunities for children. I know it's no different in what you eat, but it's actually the provision of the activities or the services that's available. (Development Partner, W) |
|  | Current considerations of gender in policy | Yes, I think the policy of having fortified iron in the products that we produce like flour that was mainly focused for our women because of the high rate of anaemia and in the past years and it is still increasing. So I think that was considered for the females. (Private Sector, W) | I think the policies that we have - it's more general. Even though, like I will say with the strategic development plan, we have some programs in there that looks at women, but overall policies are more general. (Development Partner, W) |
|  | Targeting not needed (women and men have the same needs) | I think no. I mean, priority on nutrition should be like, you know , the priority should be both genders. Why only one? (Civil Society, W) | Yeah well, I'd like to think men are always healthier, but with our anaemia stats, men are also becoming anaemic. So I think that we'd be equally affected both males and females, because the environment is the same and even in households we would be consuming the same meal. I would say that the factors affecting nutritional status, both male and females would be subjected to this same environmental factors, dietary intake, consumption patterns, purchasing power. (Govt, W) |
| 5. Enablers and barriers to the inclusion of gender in nutrition and health related policy | Barriers to the inclusion of gender | I think it will be collaborative effort. Like just safe agriculture wants it, it should work with the ministry of women. We tried to do it with agriculture. We worked with [*organisation* *name removed*] because there was report put out on gender, the key results were put out. The biggest challenge that I see amongst ministries because they see gender as a 'women' thing, hence the need for awareness and understanding. (Development Partner, W) | I'm not quite sure how you would do that. I mean, of course, generally one when one's doing kind of the communication and social mobilization and so on, that very much considers gender roles and so on, but not so much at the policy level. No, it's more of a community outreach level. (Development Partner, W) |
|  | Enablers to the inclusion of gender | Engaging community groups to getting an understanding of what the needs are. I know the food and Nutrition Centre have done quite a bit of work in the communities with their kitchen garden projects. But I guess it's important to do more. It's not to say with a women's group, but also with the men - they have a part to play. (Development Partner, W) | In terms of acknowledgement and in terms of visibility, we would hope that we are going to be able to make more evident that the role that women are playing and the different roles, of course, also the difference in terms of access, which is so key, access to formal employment or access to the different agricultural inputs, access to land, which is so key for the agricultural sector. (Development Partner, W) |
